# The impact of education on cortical thickness in amyloid-negative subcortical vascular dementia: cognitive reserve hypothesis

**DOI:** 10.1186/s13195-018-0432-5

**Published:** 2018-09-27

**Authors:** Na-Yeon Jung, Hanna Cho, Yeo Jin Kim, Hee Jin Kim, Jong Min Lee, Seongbeom Park, Sung Tae Kim, Eun-Joo Kim, Jae Seung Kim, Seung Hwan Moon, Jae-Hong Lee, Michael Ewers, Duk L Na, Sang Won Seo

**Affiliations:** 1 Pusan National University Yangsan Hospital, Pusan National University School of Medicine and Research Institute for Convergence of Biomedical Science and Technology, Yangsan, Korea; 2 Department of Neurology, Samsung Medical Center, Sungkyunkwan University School of Medicine, Seoul, Republic of Korea; 3 Neuroscience Center, Samsung Medical Center, Seoul, Korea; 4 Department of Neurology, Gangnam Severance Hospital, Yonsei University College of Medicine, Seoul, Korea; 5 Department of Neurology, Chuncheon Sacred Heart Hospital, Hallym University College of Medicine, Chuncheon, Korea; 6 Department of Biomedical Engineering, Hanyang University, Seoul, Korea; 7 Department of Radiology, Samsung Medical Center, Sungkyunkwan University School of Medicine, Seoul, Korea; 8 Department of Neurology, Pusan National University Hospital, Busan, Korea; 9 Department of Nuclear Medicine, University of Ulsan College of Medicine, Asan Medical Center, Seoul, Korea; 10 Department of Nuclear Medicine, Samsung Medical Center, Sungkyunkwan University School of Medicine, Seoul, Korea; 11 Department of Neurology, University of Ulsan College of Medicine, Asan Medical Center, Seoul, Korea; 12 Institute for Stroke and Dementia Research, Klinikum der Universität München, Ludwig-Maximilians-Universität LMU, Munich, Germany

**Keywords:** Cognitive reserve, Gray matter atrophy, Education, Subcortical vascular dementia

## Abstract

**Background:**

The protective effect of education has been well established in Alzheimer’s disease, whereas its role in patients with isolated cerebrovascular diseases remains unclear. We examined the correlation of education with cortical thickness and cerebral small vessel disease markers in patients with pure subcortical vascular mild cognitive impairment (svMCI) and patients with pure subcortical vascular dementia (SVaD).

**Methods:**

We analyzed 45 patients with svMCI and 47 patients with SVaD with negative results on Pittsburgh compound B positron emission tomographic imaging who underwent structural brain magnetic resonance imaging. The main outcome was cortical thickness measured using surface-based morphometric analysis. We also assessed the volumes of white matter hyperintensities (WMH) and numbers of lacunes as other outcomes. To investigate the correlation of education with cortical thickness, WMH volume, and number of lacunes, multiple linear regression analyses were performed after controlling for covariates, including Mini Mental State Examination, in the svMCI and SVaD groups.

**Results:**

In the SVaD group, higher education was correlated with more severe cortical thinning in the bilateral dorsolateral frontal, left medial frontal, and parahippocampal areas, whereas there was no correlation of education with cortical thickness in the svMCI group. There was no correlation between education and cerebral small vessel disease, including WMH and lacunes, in both patients with svMCI and patients with SVaD.

**Conclusions:**

Our findings suggest that the compensatory effects of education on cortical thinning apply to patients with SVaD, which might be explained by the cognitive reserve hypothesis.

## Background

Subcortical vascular dementia (SVaD) is the most common type of vascular dementia in the memory clinic setting. Unlike other types of vascular dementia showing a stepwise deterioration and fluctuating course, patients with SVaD have insidious onset and gradual progression of disease, similarly to neurodegenerative dementia [1]. SVaD is characterized by extensive cerebral small vessel disease (CSVD), such as white matter hyperintensities (WMH) and lacunes, on magnetic resonance imaging (MRI) [2]. Previous studies showed that about 30% of patients with SVaD had increased uptake of amyloid-β (Aβ) measured by carbon-11-labeled Pittsburgh compound B ([^11^C]PiB) positron emission tomography (PET) [3]. Aβ-negative patients with SVaD and those with its prodromal stage, subcortical vascular mild cognitive impairment (svMCI), showed cortical thinning prominently in the frontal and perisylvian regions, although CSVD was located predominantly in the subcortical regions [4]. In fact, cortical thinning might be considered as an important biomarker in patients with SVaD and patients with svMCI because it has been reported that CSVD was independently associated with cortical thinning, regardless of increased uptake of Aβ [5].

In previous studies, the protective effects of education reducing the risk of dementia have been shown in neurodegenerative diseases, including Alzheimer’s disease (AD) and frontotemporal dementia (FTD) [6–9]. Neuroimaging studies showed higher levels of education to be correlated with more severe neurodegeneration as measured by cortical atrophy, hypometabolism, or hypoperfusion in patients with AD dementia [10, 11], mild cognitive impairment (MCI) [12], preclinical AD [13], or FTD [14] when controlled for cognitive performance. Results from these studies have been explained by the theory of cognitive reserve [15]. Cognitive reserve refers to the ability to make efficient and flexible use of the brain for task performance, and it is estimated using education, IQ, and leisure activities [16]. In the cognitive reserve concept, individuals with higher education can cope with a larger extent of neurodegeneration before showing a level of cognitive impairment similar to that of someone with a lower level of education. Higher network efficiency and capacity (neural reserve) or compensatory functional brain differences (neural compensation) may account for such a higher reserve ability [16, 17]. Thus, according to the cognitive reserve hypothesis, protective life factors such as education mitigate the impact of brain pathology in cognition.

Beyond AD, cognitive reserve theory has not been well studied in vascular dementia. Previous studies reported that lower education levels predicted an increased risk of developing of poststroke cognitive impairment [18, 19]. In support of the hypothesis that education is related to reserve capacity in vascular dementia, it was previously shown that patients with higher education had a higher cognitive status than those with lower education despite similar degrees of subcortical hyperintensities [20]. However, these studies did not assess the influence of education on possible comorbidities such as AD pathology or on the level of brain integrity, including gray matter atrophy.

Previous studies by our group [10, 21, 22] suggested there were differences in the relationships between levels of education and cortical thickness among levels of cognition. That is, healthy control subjects showed a positive correlation between level of education and cortical thickness, whereas patients with dementia showed a negative correlation between level of education and cortical thickness. Another study by our group [23] suggested that there might be an inflection point in MCI stage. That is, the protective effects of education against cognitive decline remain in early-stage amnestic MCI and disappear in late-stage amnestic MCI. It would therefore be reasonable to expect that the correlation of education with cortical thinning might be more prominent in dementia stage rather than in MCI stage.

In the present study, we examined the correlation of education with cortical thickness and CSVD markers in patients with PiB(-) svMCI and patients with PiB(-) SVaD. We divided these patients into svMCI and SVaD groups because the cortical thinning patterns and the severity of cortical thinning and CSVD markers are different between these two groups [4]. The correlation of education might be distinct according to different vulnerable areas. On the basis of the cognitive reserve hypothesis, we predicted that both patients with SVaD and patients with svMCI would show a negative correlation between education and cortical thickness or a positive correlation between education and CSVD MRI markers when controlling for cognitive status in each group. In addition, we supposed that the correlation of education would be maximized in the SVaD group.

## Methods

### Participants

We prospectively recruited 67 patients with svMCI and 70 patients with SVaD, all of whom had been clinically diagnosed at Samsung Medical Center between September 2007 and August 2011. Patients with SVaD met the diagnostic criteria for vascular dementia as determined using criteria of the *Diagnostic and Statistical Manual of Mental Disorders, Fourth Edition* [24]. All patients with SVaD exhibited significant ischemia as determined by MRI scans, defined as a cap or band ≥ 10 mm as well as a deep white matter lesion ≥ 25 mm (a modification of the Fazekas ischemia criteria) [25]. Patients with svMCI were diagnosed using the Petersen criteria [26] with inclusion of the following modifications: (1) subjective cognitive complaints by the patient or his/her caregiver, (2) normal activities of daily living, (3) objective memory decline assessment below the 16th percentile on neuropsychological tests, (4) absence of dementia, and (5) presence of a subcortical vascular feature defined as both a focal neurological symptom/sign and significant ischemia on MRI, as for SVaD. We excluded patients with focal cortical cerebrovascular lesions, intracranial hemorrhage, territory infarctions, and hydrocephalus and those with high signal abnormalities on MRI owing to radiation injury, multiple sclerosis, vasculitis, or leukodystrophy.

In our previous studies, 32.8% (22 of 67) of patients with svMCI and 32.9% (23 of 70) of patients with SVaD were PiB-PET-positive [3, 4, 27]. We excluded PiB-positive patients with svMCI and PiB-positive patients with SVaD in the present study because we wanted to exclude the possibility that the effects of education were driven by AD. As a result, a total of 45 PiB(−) patients with svMCI and 47 PiB(−) patients with SVaD were included. We obtained written consent from each patient, and the institutional review board of Samsung Medical Center approved the study protocol.

### Neuropsychological tests

All participants underwent neuropsychological tests using a standardized neuropsychological battery, the Seoul Neuropsychological Screening Battery, for diagnostic purposes [28]. The battery comprised tests for attention, language, calculation, praxis, visuospatial/constructive function, verbal/visual memory, and frontal/executive function as previously described [29]. One patient with PiB(−) SVaD did not complete the battery, owing to severe cognitive impairment.

### Education

To thoroughly evaluate the level of education achieved by the participants, we gathered information about the number of years of formal education that they had completed. The South Korean education system is composed of elementary school (6 years), middle school (3 years), high school (3 years), and university (4 years). We confirmed the patients’ education history with their caregivers.

### [^11^C]PiB PET imaging

All patients had an [^11^C]PiB-PET scan at Samsung Medical Center or Asan Medical Center. All participants completed the same type of PET scan with a Discovery STe PET/CT scanner (GE Medical Systems, Milwaukee, WI, USA). The detailed radiochemistry profiles and scanning protocol were described in a previous study [3]. Data processing was performed using SPM version 5 in MATLAB 6.5 (MathWorks, Natick, MA, USA). To measure PiB retention, we used the cerebral cortical region/cerebellum uptake ratio, which is identical to the standardized uptake value ratios. Patients were considered PiB-positive if their global PiB retention ratio was > 2 SD greater than the mean of the normal control subjects [3].

### Acquisition of three-dimensional MRI images

Using the same 3.0-T MRI scanner (Philips 3.0 T Achieva; Philips Healthcare, Andover, MA, USA), we acquired 3D T1 turbo field echo MRI and fluid-attenuated inversion recovery (FLAIR) images from all participants as previously described [30]. Imaging parameters were as follows: T1-weighted—slice thickness, 1 mm; repetition time (TR)/echo time (TE), 4.6/9.9 ms; flip angle, 88 degrees; field of view, 24 × 24 cm; and matrix size, 240 × 240 pixels; and FLAIR—axial slice thickness, 2 mm; no gap; TR/inversion time/TE, 11,000/2800/125 ms, respectively; field of view, 24 × 24 cm; and matrix size, 256 × 256 pixels.

### Measurement of CSVD markers

We quantified WMH volume (in milliliters) on FLAIR images using an automated method as previously described [31]. WMH candidate region mask was generated on T1 images. FLAIR images were subjected to nonuniformity correction, intensity normalization, and coregistration of FLAIR and T1 images of each subject. WMH was segmented using the FMRIB Automatic Segmentation Tool (FAST) algorithm with WMH candidate region mask on FLAIR images. By using an intensity substitution method, T1 images could be classified into white matter and gray matter and localized into lobes properly. After regional parcellation, local WMH volume in four lobes was quantified. We used the total volume as a dependent variable. Lacunes were defined as lesions (≥ 3 mm and ≤ 15 mm in diameter) with low signal on T1-weighted images, high signal on T2-weighted images, and a perilesional halo on 80 axial sections of FLAIR images. The detailed measurement methods for lacunes are described in a previous paper [27]. We used the number of lacunes as a dependent variable.

### Image processing for cortical thickness measurements

Images were processed using the standard Montreal Neurological Institute anatomic pipeline. Native MRI images were registered into a standardized stereotaxic space using a linear transformation. After nonuniformities were corrected, the images were classified into white matter or gray matter using a 3D stereotaxic brain mask and the Intensity-Normalized Stereotaxic Environment for Classification of Tissues (INSECT) algorithm. The surfaces of the inner and outer cortices were automatically extracted using the Constrained Laplacian-Based Automated Segmentation with Proximities (CLASP) algorithm. Cortical thickness was defined as the Euclidean distance between the linked vertices of the inner and outer surfaces [32]. For cortical thickness analysis, diffusion smoothing with a FWHM of 20 mm was used to pixelate each map of cortical thickness, which simultaneously increased both the signal-to-noise ratio and the statistical power [33]. For global and lobar regional analyses, data of 30 normal subjects that had previously been manually categorized to lobes, and which showed high interrater reliability [34], were registered to the template. The template then took the label of maximum probability in each vertex. An individual cortical surface of a subject was registered to the precategorized template and automatically divided into frontal, temporal, parietal, and occipital lobes. Averaged values of the thickness of the whole vertex in each hemisphere and lobar region were used for our analysis. The more detailed image-processing methods are described in previous studies [30].

### Statistical analysis

The demographic and clinical differences between svMCI and SVaD were investigated using the *t* test and chi-square test. We transformed the WMH volume and the number of lacunes using square root transformations (sqrtWMH and sqrtLacune) because these variables were not normally distributed.

To investigate the correlation of education with cortical thickness and CSVD markers at the same clinical severity, multiple linear regression analyses were performed after controlling for age, sex, intracranial volume (ICV), and Mini Mental State Examination (MMSE) in both the svMCI and SVaD groups (model 1). MMSE was used as an index of the clinical severity. Because vascular risk factors affect cortical thinning [35–37], we also added hypertension, diabetes mellitus, hyperlipidemia, and smoking to the covariates of model 1 (model 2). Because WMH volume and the number of lacunes are associated with cortical thinning [5, 38], we further controlled for global cortical thickness with the covariates of model 2 in the relationship between education and CVSD markers (model 3 of Table 2). For the same reason, we further controlled for WMH and lacunes with covariates of model 2 in the relationship between education and cortical thickness (model 3 of Table 3).

## Results

### Demographics and clinical characteristics

There were no differences in the age, sex, and years of education between patients with svMCI and patients with SVaD. The prevalence of cardiovascular risk factors and the APOE ε4 allele also did not differ between patients with svMCI and patients with SVaD. However, patients with SVaD exhibited larger WMH volumes and more lacunes than patients with svMCI. Cortical thickness was thinner in patients with SVaD than in patients with svMCI (Table 1).

**Table 1:** Demographics and clinical characteristics

	PiB(−) svMCI (*n* = 45)	PiB(−) SVaD (*n* = 47)	*p* Value
Demographics			
Age, years	72.1 ± 6.6	71.9 ± 7.2	0.892
Sex, female, *n* (%)	29 (64.4)	24 (51.1)	0.194
Education, years	8.9 ± 5.3	8.5 ± 4.9	0.676
Cardiovascular risk factors, *n* (%)			
Hypertension	38 (84.4)	37 (78.7)	0.480
Diabetes mellitus	12 (26.7)	12 (25.5)	0.901
Hyperlipidemia	14 (31.1)	21 (44.7)	0.180
Smoker (current/previous/total)	0/11/40	5/12/45	0.112
*APOE* genotype^a^, *n* (%)			
ε2 allele carrier	6/45 (13.3)	5/44 (11.4)	0.778
ε4 allele carrier	5/45 (11.1)	10/44 (22.7)	0.143
Small vessel MRI markers			
WMH volume, ml	33.8 ± 17.6	41.7 ± 15.4	0.027
Lacunes, *n*	8.4 ± 8.6	20.1 ± 18.1	< 0.001
Global PiB retention ratio	1.3 ± 0.1	1.2 ± 0.1	0.085
Intracranial volume, ml	1353.7 ± 102.7	1378.1 ± 132.3	0.329
Mean cortical thickness, mm	2.8 ± 0.1	2.7 ± 0.2	< 0.001
MMSE	26.6 ± 2.4	21.5 ± 4.6	< 0.001
CDR-SOB	1.3 ± 1.0	6.0 ± 3.7	< 0.001

*Abbreviations: APOE* Apolipoprotein E, *CDR-SOB* Clinical Dementia Rating Sum of Boxes, *MMSE* Mini Mental State Examination, *MRI* Magnetic resonance imaging *PiB* Pittsburgh compound-B, *svMCI* Subcortical vascular mild cognitive impairment, *SVaD* Subcortical vascular dementia, *WMH* White matter hyperintensity

^a^*APOE* genotyping was performed in 89 of 92 participants. Values are expressed as the mean ± SD or number (%)

### Relationship between education and CSVD

In the multiple linear regression model for CSVD, after controlling for age, sex, ICV, and MMSE, there was no correlation between education level and WMH or lacunes in both the svMCI and SVaD groups (Table 2). This result was not changed after additional controlling for vascular risk factors and global cortical thickness.

**Table 2:** Multivariate analysis for the correlation of education with cerebrovascular disease markers

	sqrtWMH volume		sqrtLacunes	
	B	*p* Value	B	*p* Value
PiB(−) svMCI (*n* = 45)				
Model 1	1.590	0.251	0.053	0.249
Model 2	0.399	0.769	0.088	0.100
Model 3	0.035	0.979	0.066	0.172
PiB(−) SVaD (*n* = 47)				
Model 1	−1.114	0.393	−0.058	0.322
Model 2	−1.912	0.537	−0.051	0.430
Model 3	−1.243	0.440	−0.053	0.456

*Abbreviations: PiB* Pittsburgh Compound B, *sqrt* Square root, *svMCI* Subcortical vascular mild cognitive impairment, *SVaD* Subcortical vascular dementia, *WMH* White matter hyperintensity

Multiple linear regression was performed after controlling for the covariates as follows:

Model 1: age, sex, ICV, and MMSE control

Model 2: age, sex, ICV, MMSE, hypertension, diabetes, hyperlipidemia, and smoking control

Model 3: age, sex, ICV, MMSE, hypertension, diabetes, hyperlipidemia, smoking, and global cortical thickness control

### Relationship between education and cortical thickness

**Fig. 1:**
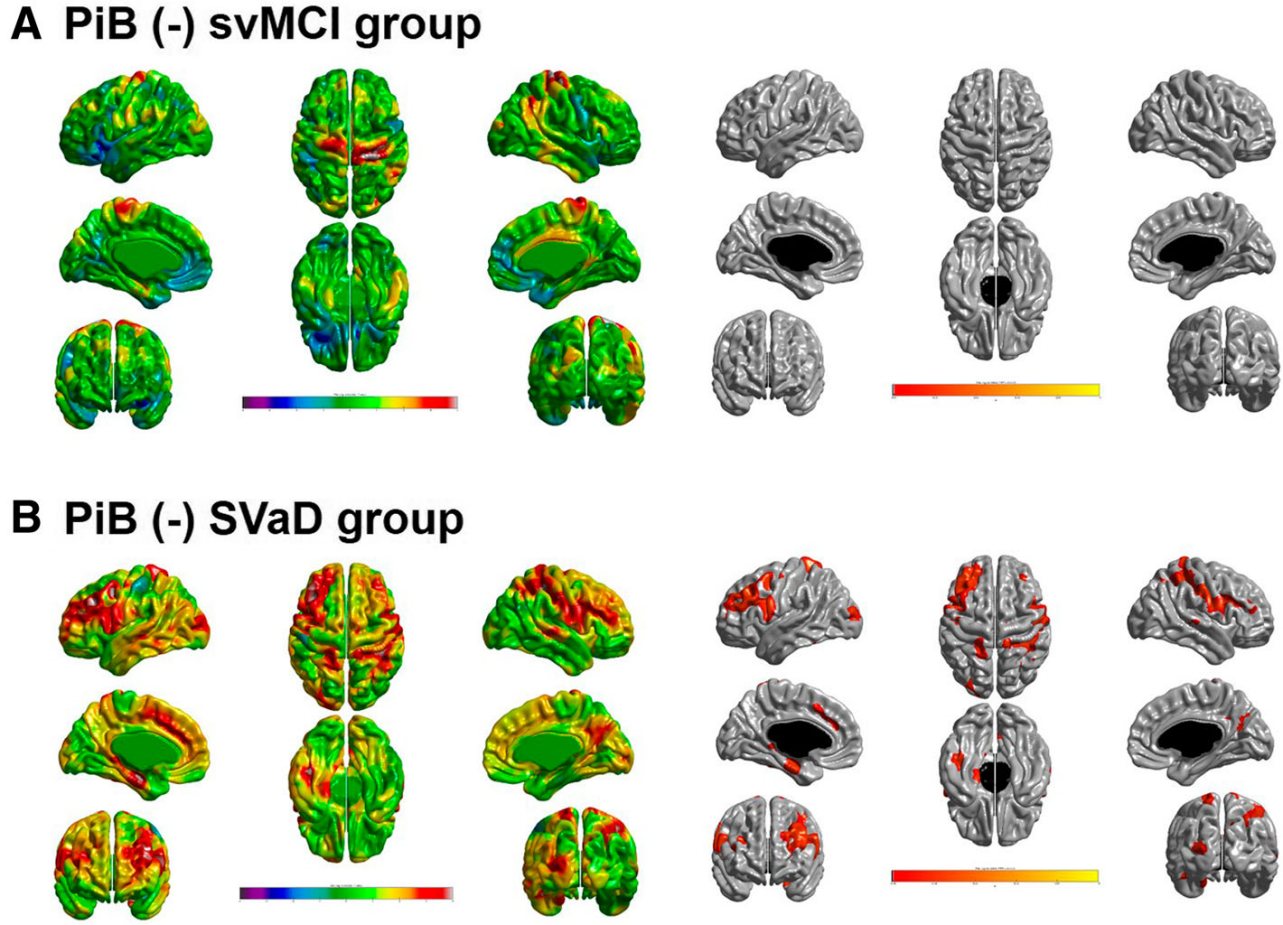
The topography of cortical thinning related to education in (**a**) PiB(−) svMCI and (**b**) PiB(−) SVaD. A negative relationship between years of education and cortical thickness was found in the bilateral dorsolateral frontal, left medial frontal, and parahippocampal areas in the PiB(−) SVaD group. *q* < 0.05 after false discovery rate correction. Adjusted for age, sex, intracranial volume, MMSE, hypertension, diabetes, hyperlipidemia, smoking, sqrtWMH, and sqrtLacune (model 3). *MMSE* Mini Mental State Examination, *PiB* Pittsburgh Compound B, *svMCI* Subcortical vascular mild cognitive impairment, *sqrt* Square root transformation, *SVaD* Subcortical vascular dementia, *WMH* White matter hyperintensities

In the multiple linear regression model for cortical thickness after controlling for age, sex, ICV, and MMSE, there was no significant relationship between education and cortical thickness in patients with svMCI (Table 3). However, there was a negative correlation of education with cortical thickness in patients with SVaD. Even when controlling for vascular risk factors, WMH (square root transformation) and number of lacunes (square root transformation), the result was not changed. The correlation between education and cortical thinning had regional specificity for the bilateral dorsolateral frontal, left medial frontal, and parahippocampal areas (Fig. 1).

**Table 3:** Multivariate analysis for the correlation of education with cortical thickness

	Cth_Global		Cth_Frontal		Cth_Temporal		Cth_Parietal		Cth_Occipital	
	B	*p* Value	B	*p* Value	B	*p* Value	B	*p* Value	B	*p* Value
PiB(−) svMCI										
Model 1	−0.004	0.352	−0.005	0.278	−0.005	0.283	−0.004	0.310	−0.001	0.800
Model 2	−0.004	0.375	−0.004	0.438	−0.004	0.398	−0.007	0.146	−0.001	0.880
Model 3	−0.001	0.823	0.001	0.859	−0.002	0.717	−0.004	0.359	0.001	0.758
PiB(−) SVaD										
Model 1	−0.016	0.028*	−0.020	0.019*	−0.014	0.115	−0.017	0.014*	−0.013	0.047*
Model 2	−0.020	0.024*	−0.023	0.024*	−0.019	0.078	−0.021	0.012*	−0.016	0.035*
Model 3	−0.020	0.029*	−0.023	0.032*	−0.020	0.065	−0.021	0.017*	−0.016	0.046*

*Abbreviations: Cth* cortical thickness, *PiB* Pittsburgh Compound B, *svMCI* Subcortical vascular mild cognitive impairment, *SVaD* Subcortical vascular dementia

* *p* < 0.05

Multiple linear regression was performed after controlling for the covariates as follows:

Model 1: age, sex, ICV, and MMSE control

Model 2: age, sex, ICV, MMSE, hypertension, diabetes, hyperlipidemia, and smoking control

Model 3: age, sex, ICV, MMSE, hypertension, diabetes, hyperlipidemia, smoking, sqrtWMH, and sqrtLacune control

## Discussion

In the present study, we investigated the correlation of education with cortical thinning and CSVD in patients with svMCI and patients with SVaD. Our main findings were as follows. First, in the SVaD group, higher education was correlated with more severe cortical thinning, whereas in the svMCI group, there was no correlation of education with cortical thickness. Second, the correlation between education and cortical thinning in patients with SVaD had regional specificity for the bilateral dorsolateral frontal, left medial frontal, and parahippocampal areas. In contrast, we did not find any correlation between education and CSVD, including WMH and lacunes, in patients with svMCI and patients with SVaD. Taken together, our findings suggested that education gives an ability to endure cortical thinning in patients with SVaD, which might be explained by the cognitive reserve hypothesis.

Our major finding was that a higher level of education was correlated with more severe cortical thinning in patients with SVaD. Our findings are consistent with a previous study based on cerebral autosomal dominant arteriopathy with subcortical infarcts and leukoencephalopathy (CADASIL), which is a genetic variant of pure vascular cognitive impairment with severe CSVD. That study demonstrated that education had a protective effect on cognitive performance in patients with vascular pathology [39]. However, there was no correlation between education and cortical thickness in patients with svMCI in our study. The results are also consistent with studies in patients with AD dementia, showing higher education levels to be associated with more cortical thinning [10], whereas these effects were not prominent in MCI stage [40, 41]. In terms of MCI, not subcortical vascular type, there are several studies with inconsistent results regarding cognitive reserve. Cognitive reserve proxies, including education, are not associated with cortical atrophy [40, 41] or functional activity [12], but cognitive reserve has a negative association with brain volumes [12]. Researchers in one study reported that higher cognitive reserve, including education, is related to more severe hypometabolism in MCI [42].

Although the correlation of education with gray matter thinning in AD but not in MCI seems perplexing, the results may be reconciled when considering the dynamic evolution of reserve effects during the course of AD. We previously observed in a longitudinal study that better-educated participants with early-stage MCI had a slower decline in cognition, whereas better-educated participants with late-stage MCI had faster rates of cognitive decline than less well-educated participants with MCI [23]. Better-educated participants with late-stage MCI were more likely to convert to AD dementia within the next 1.4 years [23]. Consistent with those results, previous longitudinal studies showed higher education to be associated with steeper cognitive decline shortly before conversion to AD [43, 44]. These results suggest that individuals with higher education may dynamically exhaust the reserve capacity until a threshold where an even steeper cognitive decline follows owing to the relatively high degree of accumulated pathology. Thus, better-educated individuals should have accrued, especially at the dementia level, much more neurodegeneration than individuals with lower education levels, whereas at the MCI level, the accrued neurodegeneration will be more subtle. Although reserve effects in prodromal AD have been demonstrated across different studies [45, 46], the neurodegeneration in svMCI may be more subtle, and thus any reserve effects may be more difficult to detect.

Unexpectedly, we did not find any correlation between the level of education and CSVD MRI markers in patients with svMCI and patients with SVaD, suggesting that high education level had no role in buffering against vascular pathology. In a previous study, higher cognitive reserve (e.g., education) was associated with more severe WMH [47]. In healthy elderly people, higher cognitive reserve protects against the cognitive deterioration related to WMH [47–49]. One study in which researchers investigated patients with CADASIL showed that education significantly influenced cognitive function in patients with mild to moderate brain pathology, including lacunes, whereas there was no effect of education on cognition in patients with severe pathology [39]. We supposed that cognitive reserve mitigates the impact of cortical thinning but not CSVD in patients with already substantial vascular pathology such as our participants. In svMCI and SVaD, CSVD burden may exceed a threshold beyond which the attenuating effect of education on cognitive function may disappear.

Our final finding was that the association between education and cortical thinning in SVaD had regional specificity for the frontal region, which is partially different from the distribution of the association in AD. There have been no studies investigating the specific brain regions related to education in patients with SVaD. Considering that patients with PiB(-) SVaD had cortical thinning prominently in the frontal and perisylvian regions, our findings suggested that the regions related to education in PiB(−) SVaD seemed to overlap with prominently involved regions in PiB(−) SVaD. In fact, a previous study based on patients with AD showed that higher education levels were found to correlate with cortical thinning prominently in the temporoparietal association region where patients with AD had characteristic cortical thinning [10]. Our findings therefore suggest that the correlation of education with cortical thickness occurs in the vulnerable regions where each type of dementia showed cortical thinning [4]. The compensatory mechanism related to specific areas has not been extensively established. Further investigation is needed. However, our findings suggested that the cognitive hypothesis might be applicable to patients with PiB(-) SVaD.

The strengths of our study are that we investigated the correlation of education with cortical thickness in patients with vascular pathologies without AD pathologies using multimodal imaging analyses. However, there are some limitations of the current study. First, we did not obtain more information on sociodemographic variables, such as occupation and leisure activities, other than the duration of education to confirm cognitive reserve. Second, we did not consider the effects of other pathologies, including other AD pathologies (soluble Aβ and neurofibrillary tangles), microinfarcts, or possible combined degenerative dementia (dementia with Lewy bodies and FTD) pathologies, which are also associated with cortical thinning. Third, the education level (mean 8.7 years) of our participants was lower than that of other cohorts (mean 15–17 years), such as the Alzheimer’s Disease Neuroimaging Initiative [41, 50]. Elderly Koreans had less opportunities for educational attainment owing to the Korean War and an increased dropout rate owing to financial reasons following the war. Finally, our study population included a large proportion of patients with significant vascular burden, which may limit the generalizability of our data to other populations.

## Conclusions

We investigated the correlation of education with cortical thickness in patients with vascular pathologies without AD pathologies using multimodal imaging analyses. Our findings suggest that the protective effect of education apply to patients with SVaD, which might be accounted for by the cognitive reserve hypothesis. It will be important to identify those functional and structural brain mechanisms that may support cognitive reserve in AD [45] and cerebrovascular disease.

## References

[1] Román GC, Erkinjuntti T, Wallin A, Pantoni L, Chui HC (2002). Subcortical ischaemic vascular dementia. Lancet Neurol.

[2] O'Brien John T, Erkinjuntti Timo, Reisberg Barry, Roman Gustavo, Sawada Tohru, Pantoni Leonardo, Bowler John V, Ballard Clive, DeCarli Charles, Gorelick Philip B, Rockwood Kenneth, Burns Alistair, Gauthier Serge, DeKosky Steven T (2003). Vascular cognitive impairment. The Lancet Neurology.

[3] Lee JH, Kim SH, Kim GH, Seo SW, Park HK, Oh SJ, Kim JS, Cheong HK, Na DL (2011). Identification of pure subcortical vascular dementia using ^11^C-Pittsburgh compound B. Neurology.

[4] Kim HJ, Ye BS, Yoon CW, Noh Y, Kim GH, Cho H, Jeon S, Lee JM, Kim JH, Seong JK (2014). Cortical thickness and hippocampal shape in pure vascular mild cognitive impairment and dementia of subcortical type. Eur J Neurol.

[5] Kim HJ, Yang JJ, Kwon H, Kim C, Lee JM, Chun P, Kim YJ, Jung NY, Chin J, Kim S (2016). Relative impact of amyloid-β, lacunes, and downstream imaging markers on cognitive trajectories. Brain.

[6] Letenneur L, Commenges D, Dartigues JF, Barberger-Gateau P (1994). Incidence of dementia and Alzheimer’s disease in elderly community residents of south-western France. Int J Epidemiol.

[7] Stern Y, Gurland B, Tatemichi TK, Tang MX, Wilder D, Mayeux R (1994). Influence of education and occupation on the incidence of Alzheimer’s disease. JAMA.

[8] Qiu C, Bäckman L, Winblad B, Agüero-Torres H, Fratiglioni L (2001). The influence of education on clinically diagnosed dementia incidence and mortality data from the Kungsholmen Project. Arch Neurol.

[9] Premi E, Grassi M, van Swieten J, Galimberti D, Graff C, Masellis M, Tartaglia C, Tagliavini F, Rowe JB, Laforce R (2017). Cognitive reserve and TMEM106B genotype modulate brain damage in presymptomatic frontotemporal dementia: a GENFI study. Brain.

[10] Seo SW, Im K, Lee JM, Kim ST, Ahn HJ, Go SM, Kim SH, Na DL (2011). Effects of demographic factors on cortical thickness in Alzheimer’s disease. Neurobiol Aging.

[11] Kidron D, Black SE, Stanchev P, Buck B, Szalai JP, Parker J, Szekely C, Bronskill MJ (1997). Quantitative MR volumetry in Alzheimer’s disease: topographic markers and the effects of sex and education. Neurology.

[12] Solé-Padullés C, Bartrés-Faz D, Junqué C, Vendrell P, Rami L, Clemente IC, Bosch B, Villar A, Bargalló N, Jurado MA (2009). Brain structure and function related to cognitive reserve variables in normal aging, mild cognitive impairment and Alzheimer’s disease. Neurobiol Aging.

[13] Ewers M, Insel PS, Stern Y, Weiner MW, Alzheimer’s Disease Neuroimaging Initiative (2013). Cognitive reserve associated with FDG-PET in preclinical Alzheimer disease. Neurology.

[14] Borroni B, Premi E, Agosti C, Alberici A, Garibotto V, Bellelli G, Paghera B, Lucchini S, Giubbini R, Perani D, Padovani A (2009). Revisiting brain reserve hypothesis in frontotemporal dementia: evidence from a brain perfusion study. Dement Geriatr Cogn Disord.

[15] Stern Y (2006). Cognitive reserve and Alzheimer disease. Alzheimer Dis Assoc Disord.

[16] Stern Y (2012). Cognitive reserve in ageing and Alzheimer’s disease. Lancet Neurol.

[17] Satz P (1993). Brain reserve capacity on symptom onset after brain injury: a formulation and review of evidence for threshold theory. Neuropsychology.

[18] Chaudhari TS, Verma R, Garg RK, Singh MK, Malhotra HS, Sharma PK (2014). Clinico-radiological predictors of vascular cognitive impairment (VCI) in patients with stroke: a prospective observational study. J Neurol Sci..

[19] Ojala-Oksala J, Jokinen H, Kopsi V, Lehtonen K, Luukkonen L, Paukkunen A, Seeck L, Melkas S, Pohjasvaara T, Karhunen P (2012). Educational history is an independent predictor of cognitive deficits and long-term survival in postacute patients with mild to moderate ischemic stroke. Stroke.

[20] Lane EM, Paul RH, Moser DJ, Fletcher TD, Cohen RA (2011). Influence of education on subcortical hyperintensities and global cognitive status in vascular dementia. J Int Neuropsychol Soc.

[21] Kim JP, Seo SW, Shin HY, Ye BS, Yang JJ, Kim C, Kang M, Jeon S, Kim HJ, Cho H (2015). Effects of education on aging-related cortical thinning among cognitively normal individuals. Neurology.

[22] Cho H, Jeon S, Kim C, Ye BS, Kim GH, Noh Y, Kim HJ, Yoon CW, Kim YJ, Kim JH (2015). Higher education affects accelerated cortical thinning in Alzheimer’s disease: a 5-year preliminary longitudinal study. Int Psychogeriatr.

[23] Ye BS, Seo SW, Cho H, Kim SY, Lee JS, Kim EJ, Lee Y, Back JH, Hong CH, Choi SH (2013). Effects of education on the progression of early- versus late-stage mild cognitive impairment. Int Psychogeriatr.

[24] Frances A, Mack AH, Ross R, First MB, Bloom FE, Kupfer DJ (1995). The DSM-IV classification and psychopharmacology. Neuropsychopharmacology: the fourth generation of progress.

[25] Fazekas F, Kleinert R, Offenbacher H, Schmidt R, Kleinert G, Payer F, Radner H, Lechner H (1993). Pathologic correlates of incidental MRI white matter signal hyperintensities. Neurology.

[26] Petersen RC (2004). Mild cognitive impairment as a diagnostic entity. J Intern Med.

[27] Lee MJ, Seo SW, Na DL, Kim C, Park JH, Kim GH, Kim CH, Noh Y, Cho H, Kim HJ (2014). Synergistic effects of ischemia and β-amyloid burden on cognitive decline in patients with subcortical vascular mild cognitive impairment. JAMA Psychiatry.

[28] Kang Y, Na DL (2003). Seoul Neuropsychological Screening Battery.

[29] Seo SW, Im K, Lee JM, Kim YH, Kim ST, Kim SY, Yang DW, Kim SI, Cho YS, Na DL (2007). Cortical thickness in single- versus multiple-domain amnestic mild cognitive impairment. Neuroimage.

[30] Kim CH, Seo SW, Kim GH, Shin JS, Cho H, Noh Y, Kim SH, Kim MJ, Jeon S, Yoon U (2012). Cortical thinning in subcortical vascular dementia with negative C-11-PiB PET. J Alzheimers Dis.

[31] Jeon S, Yoon U, Park JS, Seo SW, Kim JH, Kim ST, Kim SI, Na DL, Lee JM (2011). Fully automated pipeline for quantification and localization of white matter hyperintensity in brain magnetic resonance image. Int J Imaging Syst Technol.

[32] Kim JS, Singh V, Lee JK, Lerch J, Ad-Dab’bagh Y, MacDonald D, Lee JM, Kim SI, Evans AC (2005). Automated 3-D extraction and evaluation of the inner and outer cortical surfaces using a Laplacian map and partial volume effect classification. Neuroimage.

[33] Lerch JP, Evans AC (2005). Cortical thickness analysis examined through power analysis and a population simulation. Neuroimage.

[34] Im K, Lee JM, Yoon U, Shin YW, Hong SB, Kim IY, Kwon JS, Kim SI (2006). Fractal dimension in human cortical surface: multiple regression analysis with cortical thickness, sulcal depth, and folding area. Hum Brain Mapp.

[35] Seo SW, Lee JM, Im K, Park JS, Kim SH, Kim ST, Ahn JH, Kim MJ, Kim GH, Kim JH (2012). Cardiovascular risk factors cause cortical thinning in cognitively impaired patients: relationships among cardiovascular risk factors, white matter hyperintensities, and cortical atrophy. Alzheimer Dis Assoc Disord.

[36] Saczynski JS, Siggurdsson S, Jonsson PV, Eiriksdottir G, Olafsdottir E, Kjartansson O, Harris TB, van Buchem MA, Gudnason V, Launer LJ (2009). Glycemic status and brain injury in older individuals: the Age Gene/Environment Susceptibility–Reykjavik Study. Diabetes Care.

[37] Cho H, Kim C, Kim HJ, Ye BS, Kim YJ, Jung NY, Son TO, Cho EB, Jang H, Lee J (2016). Impact of smoking on neurodegeneration and cerebrovascular disease markers in cognitively normal men. Eur J Neurol.

[38] Tuladhar AM, Reid AT, Shumskaya E, de Laat KF, van Norden AG, van Dijk EJ, Norris DG, de Leeuw FE (2015). Relationship between white matter hyperintensities, cortical thickness, and cognition. Stroke.

[39] Zieren N, Duering M, Peters N, Reyes S, Jouvent E, Herve D, Gschwendtner A, Mewald Y, Opherk C, Chabriat H, Dichgans M (2013). Education modifies the relation of vascular pathology to cognitive function: cognitive reserve in cerebral autosomal dominant arteriopathy with subcortical infarcts and leukoencephalopathy. Neurobiol Aging.

[40] Pillai JA, McEvoy LK, Hagler DJ, Holland D, Dale AM, Salmon DP, Galasko D, Fennema-Notestine C, Alzheimer’s Disease Neuroimaging Initiative (2012). Higher education is not associated with greater cortical thickness in brain areas related to literacy or intelligence in normal aging or mild cognitive impairment. J Clin Exp Neuropsychol.

[41] Pettigrew C, Soldan A, Zhu Y, Wang MC, Brown T, Miller M, Albert M, BIOCARD Research Team (2017). Cognitive reserve and cortical thickness in preclinical Alzheimer’s disease. Brain Imaging Behav.

[42] Garibotto V, Borroni B, Kalbe E, Herholz K, Salmon E, Holtoff V, Sorbi S, Cappa SF, Padovani A, Fazio F, Perani D (2008). Education and occupation as proxies for reserve in aMCI converters and AD: FDG-PET evidence. Neurology.

[43] Hall CB, Derby C, LeValley A, Katz MJ, Verghese J, Lipton RB (2007). Education delays accelerated decline on a memory test in persons who develop dementia. Neurology.

[44] Yu L, Boyle P, Wilson RS, Segawa E, Leurgans S, De Jager PL, Bennett DA (2012). A random change point model for cognitive decline in Alzheimer’s disease and mild cognitive impairment. Neuroepidemiology.

[45] Franzmeier N, Duering M, Weiner M, Dichgans M, Ewers M, Alzheimer’s Disease Neuroimaging Initiative (2017). Left frontal cortex connectivity underlies cognitive reserve in prodromal Alzheimer disease. Neurology.

[46] Morbelli S, Perneczky R, Drzezga A, Frisoni GB, Caroli A, van Berckel BN, Ossenkoppele R, Guedj E, Didic M, Brugnolo A (2013). Metabolic networks underlying cognitive reserve in prodromal Alzheimer disease: a European Alzheimer Disease Consortium project. J Nucl Med.

[47] Brickman AM, Siedlecki KL, Muraskin J, Manly JJ, Luchsinger JA, Yeung LK, Brown TR, DeCarli C, Stern Y (2011). White matter hyperintensities and cognition: testing the reserve hypothesis. Neurobiol Aging.

[48] Dufouil C, Alperovitch A, Tzourio C (2003). Influence of education on the relationship between white matter lesions and cognition. Neurology.

[49] Nebes RD, Meltzer CC, Whyte EM, Scanlon JM, Halligan EM, Saxton JA, Houck PR, Boada FE, DeKosky ST (2006). The relation of white matter hyperintensities to cognitive performance in the normal old: education matters. Neuropsychol Dev Cogn B Aging Neuropsychol Cogn.

[50] Petersen RC, Aisen PS, Beckett LA, Donohue MC, Gamst AC, Harvey DJ, Jack CR, Jagust WJ, Shaw LM, Toga AW (2010). Alzheimer’s Disease Neuroimaging Initiative (ADNI): clinical characterization. Neurology.

